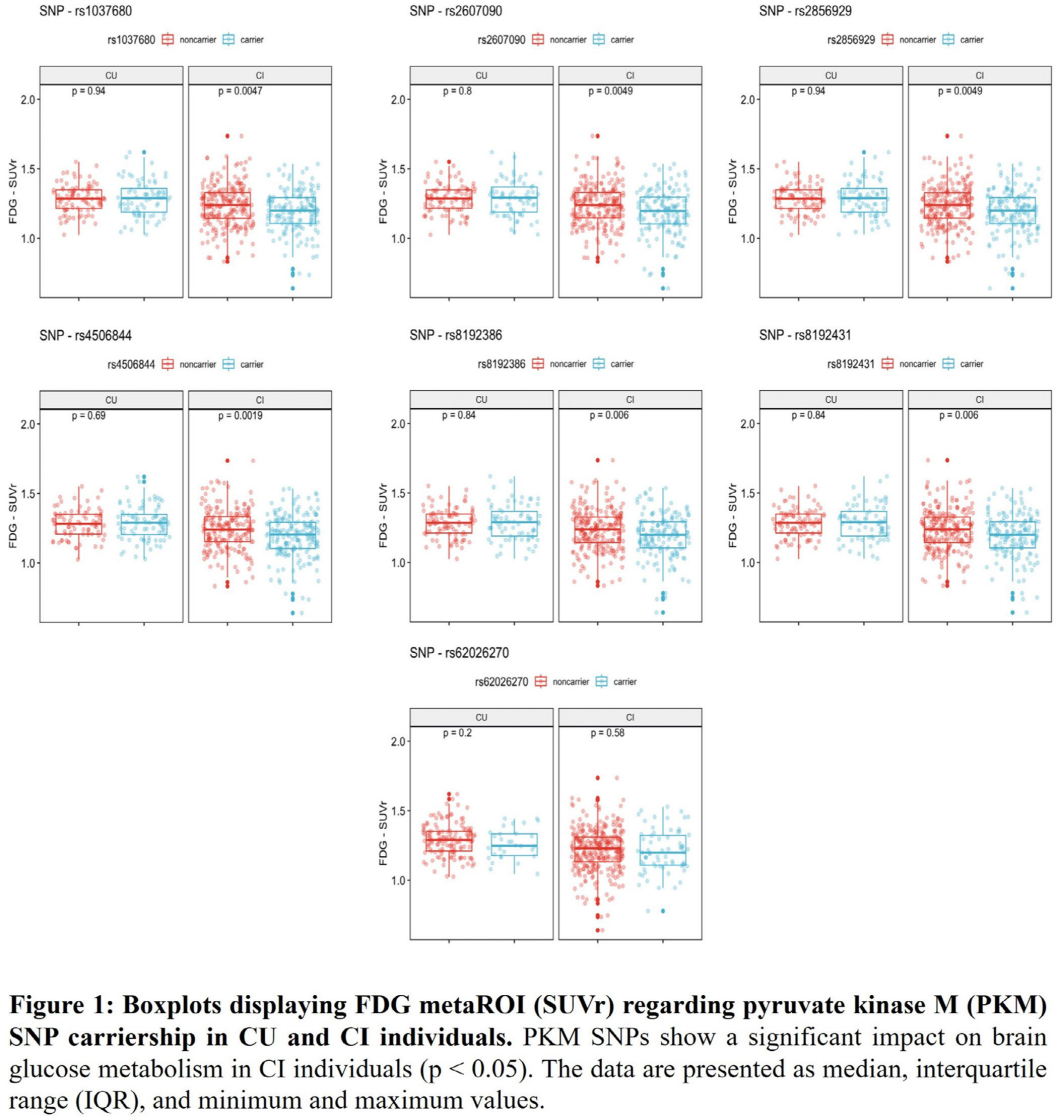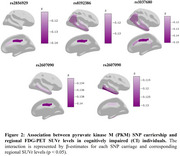# Pyruvate kinase M (PKM) single nucleotide polymorphisms are related to glucose hypometabolism in cognitively impaired individuals

**DOI:** 10.1002/alz70856_105616

**Published:** 2026-01-07

**Authors:** Lorenzo Celia Ferreira, Rodrigo Sebben Paes, Christian Limberger, Gabriela Mantovani Baldasso, Marco Antônio De Bastiani, Eduardo R. Zimmer

**Affiliations:** ^1^ Universidade Federal do Rio Grande do Sul, Porto Alegre, Rio Grande do Sul, Brazil; ^2^ Brain Institute of Rio Grande do Sul (InsCer), PUCRS, Porto Alegre, Rio Grande do Sul, Brazil; ^3^ McGill Centre for Studies in Aging, Montreal, QC, Canada

## Abstract

**Background:**

Brain glucose hypometabolism is associated with neurodegeneration in Alzheimer's disease (AD). Individuals at risk for AD can exhibit reduced glucose metabolism in brain regions typically affected by AD pathology, even years before the diagnosis. However, the impact of glycolytic metabolism dysfunction on AD progression remains to be fully elucidated. Herein, we investigated the impact of single nucleotide polymorphisms (SNPs) in the pyruvate kinase M (PKM) gene ‐ a key glycolytic enzyme ‐ on brain glucose metabolism across AD stages.

**Method:**

We analyzed 203 cognitively unimpaired (CU) and 416 cognitively impaired (CI) individuals from ADNI. Seven SNPs (rs62026270, rs2856929, rs8192431, rs8192386, rs2607090, rs1037680, and rs4506844) related to the PKM gene were identified. We examined the association between SNP carriership and brain glucose metabolism in FDG metaROI using linear mixed‐effect models, adjusted for age, sex, APOE4, amyloid, and tau status (*p* < 0.05). We also performed ROI‐wise correlation analysis to investigate the effect of SNP carriership on FDG regional SUVr levels.

**Result:**

We found that six SNPs are significantly associated with reduced brain glucose metabolism in CI individuals (β = ‐0.21 to ‐0.23, *p* < 0.05), with no effect presented in CU individuals (Figure 1). Glucose hypometabolism was particularly evident in typical AD hypometabolic regions, including the entorhinal, temporal, cingulate, and inferior parietal cortex (Figure 2).

**Conclusion:**

Our findings indicate that SNPs in the PKM gene may influence glucose metabolism in CI individuals. The reduced FDG‐PET signal, especially in regions linked to AD vulnerability, suggests these genetic variants may contribute to the hypometabolism associated with AD progression. This reinforces the idea that glucose hypometabolism is not just a consequence of neurodegeneration but may play a critical role in disease progression. Future research should investigate how these SNPs affect metabolic efficiency to identify new intervention targets for AD‐risk populations.